# Effects of *bacillu*s on continuous cropping of sugar beets and their rhizosphere microbial community

**DOI:** 10.1038/s41598-025-30744-5

**Published:** 2025-12-21

**Authors:** Yanchun Sun, Qun Song, Zenghao Wang, Youkai Gao, Liuli Wei, Yuguang Wang, Rui Chen, Gui Geng

**Affiliations:** College of Advanced Agriculture and Ecological Environment, Heilongjiang University, Harbin, 150000 China

**Keywords:** Bacterial agents, Successive cropping obstacle, Rhizosphere microorganisms, Microbial diversity, Ecology, Ecology, Environmental sciences, Microbiology, Plant sciences

## Abstract

Sugar beet is a vital sugar-producing crop, and continuous cropping poses a significant threat to its growth, leading to a decline in yield and quality. This study aimed to investigate the effects of two bacterial agents, *Bacillus subtilis* and *Bacillus mucilaginosus*, on the growth, soil physicochemical properties, and rhizosphere microbial community of sugar beet seedlings. We employed pot experiments and amplicon sequencing to analyze the impact of applying two different *Bacillus* agents on the microbial community structure in the rhizosphere soil of continuously cropped sugar beet and explore the microbial composition, environmental driving factors, and potential functions present within the microbial communities. The results showed that both *Bacillus* agents and their combination significantly promoted the growth of continuous cropping sugar beet seedlings, reaching or even surpassing the levels observed in crop rotation, improved soil pH, and enhanced soil environment. High-throughput sequencing analysis of the rhizosphere soil revealed that all *Bacillus* treatments induced changes in the diversity and structural composition of the rhizosphere microbial community, and significantly increased the relative abundance of Proteobacteria, thereby enriching beneficial microorganisms such as *Pseudomonas*, *Novosphingobium*, and *Sphingomonas* compared with that in the control group. Additionally, the application of *Bacillus* inoculants significantly enhanced the nitrate respiration, nitrogen respiration, and chitinolytic functions. These two bacterial agents optimized soil physicochemical properties and improved the rhizosphere soil microbial community structure, promoting sugar beet seedling growth and effectively mitigating the negative effects of continuous cropping.

## Introduction

Sugar beet (*Beta vulgaris* L.) is a biennial herbaceous dicotyledonous plant belonging to the family Chenopodiaceae. As a globally significant vegetable crop, it contributes to 30% of the world’s sugar production and is one of the major sugar-producing crops in China and worldwide^1^. Beyond sugar production, sugar beet is also used to produce ethanol, glycerol, citric acid, and other light industrial products. Additionally, it serves as a valuable feed source, offering high comprehensive utilization value^2,3^. Compared with sugarcane, sugar beet exhibits greater drought tolerance, cold resistance, and salt–alkali tolerance, demonstrating strong stress resistance and adaptability to adverse environmental conditions^4^. Sugar beet is cultivated worldwide, primarily in Russia, North America, France, Germany, and parts of northern China^5^. The continuous reduction in arable land has made continuous cropping a common farming practice in global agriculture. Continuous cropping refers to the repeated cultivation of the same crop in the same field over successive seasons^6^. Studies have shown that this practice can lead to various issues, including changes in soil enzyme activity, deterioration of soil physicochemical properties^7^, imbalances in soil microbial populations^8^, and increased soil-borne diseases. As a deep-rooted crop, sugar beet consumes a substantial amount of soil nutrients and is highly sensitive to continuous cropping. Prolonged continuous cultivation can lead to reduced sugar content, lower yields, and increased incidence of pests and diseases^9^.

A healthy soil environment is essential for the normal growth of sugar beet and is a prerequisite for achieving high sugar content and yield. Soil quality is influenced by not only its physicochemical properties but also its microbial characteristics^10^. Soil microorganisms are a fundamental component of the soil ecosystem, playing a key role in soil formation, fertility maintenance, and plant growth. They are considered key indicators of soil quality^11^. The rhizosphere, as a critical soil region surrounding plant roots, harbors microorganisms closely linked to plant growth, development, and nutrient exchange^12–14^. These microbial communities interact with plants, participate in essential nutrient cycles, and regulate most soil ecological processes, playing an irreplaceable role in maintaining plant health and soil carbon cycling^15–17^. Additionally, plant growth stages influence the structure and dynamics of the rhizosphere microbial community^18^. Soil enzymes are produced by soil microorganisms, and the changes in their activity not only reflect shifts in the microbial community but also play a crucial role in soil nutrient cycling^19,20^. Therefore, soil, microorganisms, and plants are closely interconnected, significantly influencing agricultural productivity; microbial abundance and diversity, in particular, are crucial for soil fertility and plant development^21,22^.

The widespread use of chemical fertilizers has profoundly affected the ecological functions of the soil microenvironments in agricultural ecosystems. Over the past few decades, chemical fertilizers have been extensively applied to enhance soil nutrient availability and increase crop yields. However, excessive use of chemical fertilizers has led to serious problems, such as soil degradation, reduced organic matter, and negative impacts on soil microbial diversity^23,24^. In contrast, microbial fertilizers, which contain active microorganisms and their metabolites, can effectively improve soil fertility, nutrient availability, and overall growth environments; they promote plant growth and development, enhance resistance to pests and diseases, and unlock soil potential to improve agricultural yield and quality^25,26^. *Bacillus* species, widely present in soil, can produce various metabolites that promote plant growth and protect plants from pathogens^27^. They are among the most effective biocontrol agents, offering an eco-friendly alternative to synthetic fungicides^28^. Additionally, *Bacillus* species can alter soil enzyme activity, improving soil quality and nutrient availability^29,30^. Studies have shown that the members of the *Bacillus* genus exhibit broad-spectrum antagonistic activity against various soil-borne pathogens; artificial inoculation with *Bacillus* strains significantly enhances the growth and yield of various crops^31,32^. For example, *Bacillus subtilis* strain PTS-394 promotes plant growth and suppresses soil-borne diseases in tomatoes^33^, whereas *B. subtilis* T6-1 significantly alleviates continuous cropping stress in poplar by improving the rhizosphere microbial community structure and inhibiting the growth of pathogenic fungi^34^. Furthermore, *Bacillus mucilaginosus* can promote alfalfa growth and regulate soil properties^35^. Given these benefits, investigating the effects of *Bacillus* species on the rhizosphere soil properties and microbial community structure of continuously cropped sugar beet is crucial for mitigating continuous cropping-related challenges and achieving sustainable, green cultivation of sugar beet.

Currently, 16S and internal transcribed spacer (ITS) amplicon sequencing have become essential tools for studying microbial community composition in environmental samples^10^. This study used pot experiments and high-throughput sequencing of 16S rRNA and ITS amplicons for investigating the effects of *B. subtilis* and *B. mucilaginosus* on the growth and development of continuously cropped sugar beet seedlings. Additionally, the study analyzed the changes in the physicochemical properties of rhizosphere soil and shifts in the composition and functional diversity of the rhizosphere soil microbial communities. The main objective of the study was to determine the influence of these two *Bacillus* species on continuous cropping stress in sugar beet and examine the effects of the composition and diversity of bacterial and fungal communities in its rhizosphere soil. Further, the study aimed to evaluate the correlations between changes in rhizosphere soil microorganisms and *Bacillus* application, and predict the changes in the ecological functions of these microorganisms in continuously cropped sugar beet rhizosphere soil. Ultimately, this study sought to provide a theoretical basis for mitigating continuous cropping challenges in sugar beet and offer valuable insights for developing improved agricultural management strategies.

## Materials and methods

### Materials and experimental design

The sugar beet variety “KWS1260” from KWS Company (Einbeck, Germany) was selected as the plant material. The *B. mucilaginosus* powder (5 billion viable bacteria per gram) and *B. subtilis* powder (50 billion viable bacteria per gram) were supplied by Beihai Qiangxing Biotechnology Co., Ltd. Soil samples were collected from the black soil region of Hulan District, Harbin City, Heilongjiang Province (latitude: 46°00′14′′N, longitude: 126°38′49′′E). The soil samples were from fields with 2 years of continuous sugar beet cropping. The experiment was conducted in the soil light culture room of the agricultural building at Heilongjiang University. A pot experiment was designed with five treatment groups: noncontinuous cropping soil as the positive control (NC), continuous cropping soil for 2 years as the control (CK), continuous cropping soil treated with *B. subtilis* powder at an application rate of 1 g/kg (CK + K), continuous cropping soil treated with *B. mucilaginosus* powder at an application rate of 1 g/kg (CK + J), and continuous cropping soil treated with a combination of *B. subtilis* and *B. mucilaginosus* powder, each applied at the rate of 0.5 g/kg (CK + KJ). Each treatment had eight replicates. At the start of the experiment, the required bacterial agents were mixed into the soil using the soil-mixing method, and the sugar beet seeds (KWS1260) were sown in pots containing 900 g of soil. Each pot retained five seedlings for subsequent experiments. During the seedling growth period, the light intensity was maintained at 700 μmol·m^–2^·s^–1^, with a 14-h light/10-h dark cycle. The temperature was set at 28 °C ± 1 °C during the day and 21 °C ± 1 °C at night, with a relative humidity of 40–50%.

### Soil sampling

Soil sampling was conducted on the 28th day. For non-rhizosphere soil sampling, three replicates were randomly selected from each treatment group, and samples were collected using the five-point sampling method. The three replicates were mixed and recorded as one sample. The collected non-rhizosphere soil samples were air-dried and passed through a 2-mm sterile sieve for determining soil properties and enzyme activities. For rhizosphere soil sampling, three uniformly growing sugar beet seedlings were randomly selected. A sterile scalpel was used to cut the roots of the sugar beet seedlings, and excess non-rhizosphere soil was shaken off to ensure experimental rigor. The beetroots were placed in a 50-mL sterile tube containing 25 mL of 10 mM sterile phosphate-buffered saline solution (w/v) and oscillated at 180 rpm at 4 °C for 5 h to separate the rhizosphere soil from the root surface. The samples were then centrifuged at 3200 g at 4 °C for 15 min, and the roots were removed to obtain the rhizosphere soil. Each treatment had five replicates, and the rhizosphere soil samples were stored at –80 °C for high-throughput sequencing.

### Plant growth index measurement

Plant growth was observed and recorded through random sampling of uniformly growing sugar beet plants. Plant height and root length were measured using a tape measure from the growth point to the tip of the longest leaf, with five replicates per treatment. The stem diameter was measured at the base of the first stem node using a Vernier caliper (0.02 precision), with five replicates per treatment. Leaf and root areas were scanned using an Epson scanner (Anlie-3000C; Lincoln Li-Cor Biosciences Inc., CA, USA) and analyzed using WinRHIZO software (Beijing Ecotech Co., Ltd., China), with three replicates per treatment. The fresh weight of aboveground plant parts was measured using an electronic balance (model: 5001; Hangzhou Wante Weighing Apparatus Co., Ltd.) after wiping off surface moisture and dust, with five replicates per treatment. Five sugar beet plants were randomly selected from each treatment, and their weight was measured immediately. Dry weight was measured by first drying the plant samples at 105 °C for 30 min, followed by drying at 75 °C until a constant weight was achieved. The dried samples were then weighed using an electronic balance (Shanghai Liangping Instrument Co., Ltd., China), with five replicates per treatment.

### Analysis of soil properties and enzyme activity

Soil ammonium nitrogen (AN) was determined using the indophenol blue colorimetric method, whereas soil nitrate nitrogen (NO_3_-N) was assessed using the dual-wavelength method. Soil available phosphorus (AP) was measured using the NaHCO_3_ extraction–molybdenum antimony colorimetric method. Soil available potassium (AK) and trace elements were extracted using ammonium acetate and directly measured using a flame photometer (Shanghai Yuan Analysis Instrument Co., Ltd.). Soil pH was determined using a potentiometer (Shanghai Jingke Instrument Co., Ltd.) at a water-to-soil ratio of 5:1. Soil electrical conductivity (EC) was measured using a conductivity meter (Shanghai Jingke Instrument Co., Ltd.) with the same water-to-soil ratio of 5:1. Soil enzyme activities were assessed as follows: urease (URE) activity was determined using the indophenol blue colorimetric method, phosphatase (PHO) activity was measured using the phenyl phosphate disodium colorimetric method, sucrase (SUC) activity was determined using the 3,5-dinitrosalicylic acid colorimetric method, and catalase (CAT) activity was analyzed using the potassium permanganate titration method^36^. All soil samples were measured in triplicate, and soil extracellular enzyme activities were measured with inorganic controls included for each treatment.

### Amplicon sequencing and analysis

For amplicon sequencing and analysis, total DNA from the microbial community was extracted using the cetyltrimethylammonium bromide method. DNA quality was evaluated using 2% agarose gel electrophoresis, whereas concentration and purity were determined using AMPure XT beads (Beckman Coulter Genomics, MA, USA) and Qubit (Invitrogen, USA, CA, 90,720). Bacterial polymerase chain reaction (PCR) amplification was performed using the 341F and 805R primers to amplify the V3–V4 region of the 16S rRNA gene. Fungal ITS amplification was performed using the ITS1F and ITS2R primers^37^. PCR products were recovered using 2% agarose gel electrophoresis and purified using the AMPure XT bead recovery kit. Finally, the concentration and purity of the recovered products were assessed using the Agilent 2100 Bioanalyzer (Agilent, USA, CA, 95,054) and the Illumina library quantification kit (Kapa Biosciences, MA, USA). The 16S and ITS high-throughput sequencing was performed by LC-Bio (Hangzhou, China).

The raw sequencing data were quality-controlled using the fqtrim software (v0.94, http://ccb.jhu.edu/software/fqtrim/) to remove adapter sequences and low-quality bases. Reads were assembled, and Vsearch software (v2.3.4, https://github.com/torognes/vsearch) was used for operational taxonomic unit (OTU) clustering at a 97% similarity threshold, with chimeras removed. The representative OTU sequences were annotated using the SILVA(v138, https://www.arb-silva.de/documentation/release138/) and UNITE(v8.0, https://unite.ut.ee/) databases at a confidence threshold of 0.7 to obtain species taxonomic annotation results^38,39^. Sequences corresponding to chloroplasts and mitochondria were removed, and OTU data were normalized using the minimum sampling method^40^.

### Statistical analysis

Statistical analyses of plant and soil parameters were performed using IBM SPSS 26 (https://www.ibm.com/cn-zh/spss) statistical software (SPSS Inc., CT, USA) for one-way analysis of variance. The normality of the data was assessed using the Shapiro–Wilk test (*P* > 0.05), whereas the homogeneity of variance was examined using Levene’s test (*P* > 0.05). Based on the outcomes of the homogeneity of variance analysis, Duncan’s multiple range test (*P* > 0.05) was employed when the significance level exceeded 0.05. In contrast, the Kruskal–Wallis nonparametric test (*P* < 0.05) was applied when the significance level fell below 0.05. All statistical analyses were conducted with a 95% confidence interval. The Mothur software^41^ (v1.21.1, https://mothur.org/wiki/download_mothur/) was used to calculate α-diversity, including the Chao1 and Simpson indices. Duncan’s multiple range test was used to assess the differences between continuous cropping and bacterial agent–treated sugar beet soil samples. Non-metric multidimensional scaling (NMDS) was used to calculate the Bray–Curtis distance matrix and visualize it to examine the β-diversity of microbial community functions. Linear discriminant analysis (LDA) effect size (LEfSe) was used to identify microbial taxonomic biomarkers^42^. The “ggcor” package in R (version 4.1.3) was used for Spearman correlation analysis, whereas Manter tests were conducted to evaluate the relationship between microbial community composition and environmental factors^43^. Spearman correlations were calculated using the R package to explore correlations among all microorganisms, with a Spearman correlation coefficient r value of 0.7 and *P* < 0.01. Other analyses were performed using R software (version 4.1.3) and the LC-Bio cloud platform.

## Results

### Soil properties and growth indicators

Different *Bacillus* treatments significantly affected various growth indicators of continuous cropping sugar beet seedlings (Table 1). Compared with the NC group, the CK group showed a significant reduction in terms of plant Height, stem diameter (SD), aboveground dry weight (DW), root dry weight (RDW), root surface area (RSA), and leaf surface area (LSA), only in root length (RL) was there no significant difference observed. This result confirms the inhibitory effect of continuous cropping on sugar beet growth. The three bacterial inoculation treatment groups also exhibited significant differences compared with the CK group. Specifically, all three *Bacillus*-treated groups (CK + K, CK + J, and CK + KJ) significantly enhanced sugar beet seedling Height, SD, DW and LSA, exceeding those in both the CK and NC groups, with the CK + KJ group showed the most significant increase, with an average plant Height of 16.73 cm and an average SD of 5.261 cm, marking increments of 29.39% and 35.84% over the CK group, respectively. And the CK + J group showed the most significant increase in DW and LSA. Additionally, all three *Bacillus*-treated groups (CK + K, CK + J, and CK + KJ) showed significantly higher RDW, RL and RSA compared with the CK group, and no significant variation compared with the NC group. These results indicated that both *Bacillus* agents and their combination significantly promoted the growth of continuous cropping sugar beet seedlings, reaching or even surpassing the levels observed in crop rotation.

**Table 1:** Plant indicators of different groups.

Group	NC	CK	CK + K	CK + J	CK + KJ
Height (cm)	14.27 ± 0.196c	12.93 ± 0.256d	14.65 ± 0.220c	15.53 ± 0.105b	16.73 ± 0.161a
SD (cm)	4.214 ± 0.386c	3.873 ± 0.331d	4.165 ± 0.264c	4.748 ± 0.205b	5.261 ± 0.328a
DW (g)	0.410 ± 0.021c	0.177 ± 0.007d	0.550 ± 0.051b	0.663 ± 0.029a	0.560 ± 0.006b
RDW (g)	0.213 ± 0.021a	0.127 ± 0.015b	0.253 ± 0.032a	0.277 ± 0.047a	0.217 ± 0.046a
RL (cm)	24.66 ± 2.499ab	22.31 ± 3.056b	24.87 ± 1.333a	24.57 ± 1.545ab	23.61 ± 2.057ab
RSA (cm^2^)	65.80 ± 4.821ab	39.33 ± 5.239c	60.98 ± 7.343b	83.98 ± 5.869a	81.88 ± 4.642a
LSA (cm^2^)	63.35 ± 4.353c	32.35 ± 1.239d	74.83 ± 8.101c	102.3 ± 1.637a	91.58 ± 1.175b

NC, Noncontinuous cropping soil; CK, continuous cropping soil for 2 years as the control; CK + K, continuous cropping soil with *B. subtilis*; CK + J, continuous cropping soil with *B. mucilaginosus*; CK + KJ, continuous cropping soil with a combination of *B. subtilis* and *B. mucilaginosus*. Height, Plant height; SD, stem diameter; DW, aboveground dry weight; RDW, root dry weight; RL, root length; RSA, root surface area; LSA, leaf surface area. The values in the table are presented as the average ± standard deviation (*n* = 5). Different lowercase letters in the same row indicate significant differences (*P* < 0.05; Duncan and Kruskal–Wallis tests).

Applying two *Bacillus* inoculants and their combination altered the physicochemical properties and enzyme activities of continuous cropping soil (Table 2). The pH values in all *Bacillus*-inoculated treatment groups were significantly higher than those in the CK group, with the CK + J group showed the most pronounced effect, achieving a pH level close to that of NC. The measurements of ammonium nitrogen (AN), available phosphorus (AP), and available potassium (AK) in the soil after *Bacillus* treatments revealed three distinct outcomes. Compared with the CK, neither the individual *Bacillus* inoculants (CK + K, CK + J) nor their combination (CK + KJ) exhibited significant differences in the AN content. However, the AP content increased significantly by 12.18%, 7.99%, and 19.26% in the treated groups, with the CK + KJ group exhibiting a significantly higher increase than both CK + K and CK + J groups. Additionally, both AN and AP contents were lowest in the NC group, with a significant difference from the other four groups. In contrast, the AK content in *Bacillus*-inoculated groups was considerably lower than in the CK group, and close to the NC group. These results suggested that *B. subtilis* and *B. mucilaginosus* could promote an increase in soil AP content, with a synergistic effect between the two *Bacillus* strains, while having no effect on AN content and reducing AK content. Differences were also observed in soil enzyme activities among *Bacillus* treatments. Compared with the CK group, the NC group and *Bacillus*-treated groups exhibited reduced Catalase (CAT) activity, with the combined *Bacillus* inoculant treatment (CK + KJ) causing the most significant decrease. Phosphatase (PHO) activity did not differ significantly between groups, whereas urease (URE) activity increased in the *Bacillus*-inoculated groups, particularly in the CK + KJ group, which showed a 19% increase over the CK group. Additionally, sucrase (SUC) activity in all three *Bacillus*-inoculated treatment groups was increased, with the CK + K group significantly higher than that in the CK group. These findings suggested that both *Bacillus* strains could promote specific soil enzyme activities.

**Table 2:** Physicochemical properties and enzyme activities of soil in different groups.

Grope	NC	CK	CK + K	CK + J	CK + KJ
pH	6.98 ± 0.008a	6.42 ± 0.015c	6.9 ± 0.015b	6.94 ± 0.038ab	6.89 ± 0.026b
AN(mg/kg)	4.21 ± 0.407b	5.35 ± 0.159a	5.54 ± 0.242a	5.17 ± 0.121a	5.58 ± 0.358a
AP(mg/kg)	76.05 ± 2.481d	129.03 ± 4.342c	144.74 ± 2.261b	139.35 ± 2.379b	153.88 ± 0.812a
AK(mg/kg)	315.06 ± 4.033c	603.15 ± 9.272a	331.82 ± 2.640bc	272.38 ± 5.280d	347.07 ± 4.033b
Catalase(μmol/d/g)	38.04 ± 1.318b	43.45 ± 1.026a	33.74 ± 3.301b	33.74 ± 5.990c	26.47 ± 1.710c
Phosphatase(nmol/d/g)	1.88 ± 0.343a	2.10 ± 0.223a	2.67 ± 0.374a	2.39 ± 0.162a	2.32 ± 0.079a
Urease(mg/d/kg)	501.34 ± 16.852b	527.88 ± 41.605b	577.80 ± 4.696ab	567.46 ± 14.216ab	628.18 ± 26.383a
Sucrase(mg/g)	52.47 ± 0.199ab	50.61 ± 1.428b	55.50 ± 0.207a	55.08 ± 0.406ab	54.19 ± 0.073ab

NC, Noncontinuous cropping soil; CK, continuous cropping soil for 2 years as the control; CK + K, continuous cropping soil with *B. subtilis*; CK + J, continuous cropping soil with *B. mucilaginosus*; CK + KJ, continuous cropping soil with a combination of *B. subtilis* and *B. mucilaginosus*. pH, Soil acidity/alkalinity; AN, ammonium nitrogen; AP, available phosphorus; AK, available potassium. The values in the table are presented as the average ± standard deviation (*n* = 5). Different lowercase letters in the same row indicate significant differences (*P* < 0.05; Duncan and Kruskal–Wallis tests).

### Alpha diversity of microbial communities

**Fig. 1:**
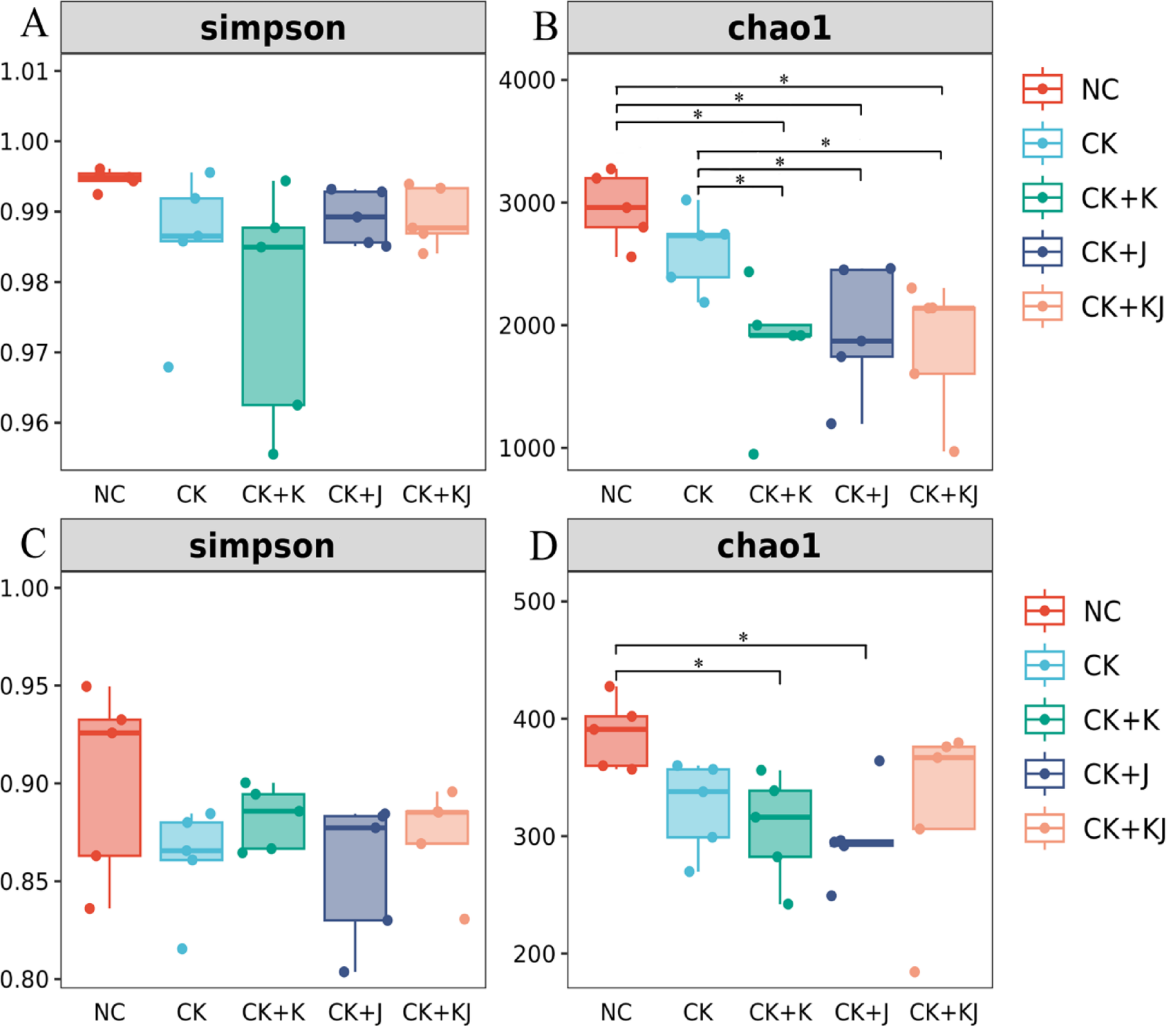
Alpha diversity of bacterial and fungal communities. (**A** and **B**) Bacterial alpha diversity; (**C** and **D**) fungal alpha diversity. Simpson index (**A** and **C**); Chao1 index (**B** and **D**). “*” indicates significance at *P* < 0.05. NC, Noncontinuous cropping soil; CK, continuous cropping soil for 2 years as the control; CK + K, continuous cropping soil with *B. subtilis*; CK + J, continuous cropping soil with *B. mucilaginosus*; CK + KJ, continuous cropping soil with a combination of *B. subtilis* and *B. mucilaginosus*.

Alpha diversity indices are critical parameters for assessing species diversity within communities. This study used Chao1 and Simpson indices to evaluate the alpha diversity of rhizosphere microbial communities among noncontinuous cropping soil, continuous cropping soil and *Bacillus*-treated soil (Fig. 1). The CK treatment showed mildly reductions in the richness and diversity of bacterial and fungal communities compared with NC treatment group (Fig. 1A–D). Compared with CK, *Bacillus* treatments significantly reduced the bacterial richness of the rhizosphere soil (Chao1) but had minimal impact on bacterial diversity (Simpson index) (Fig. 1A and B). Meanwhile, *Bacillus* treatments showed no significant effect on the diversity of the soil fungal community. However, both the CK + K and CK + J treatment groups demonstrated moderate reductions in the richness of soil fungal community to some extent (Fig. 1C and D). Overall, *Bacillus* inoculants mildly reduced the richness of bacterial and fungal communities but did not significantly affect their diversity.

### Beta diversity of microbial communities

**Fig. 2:**
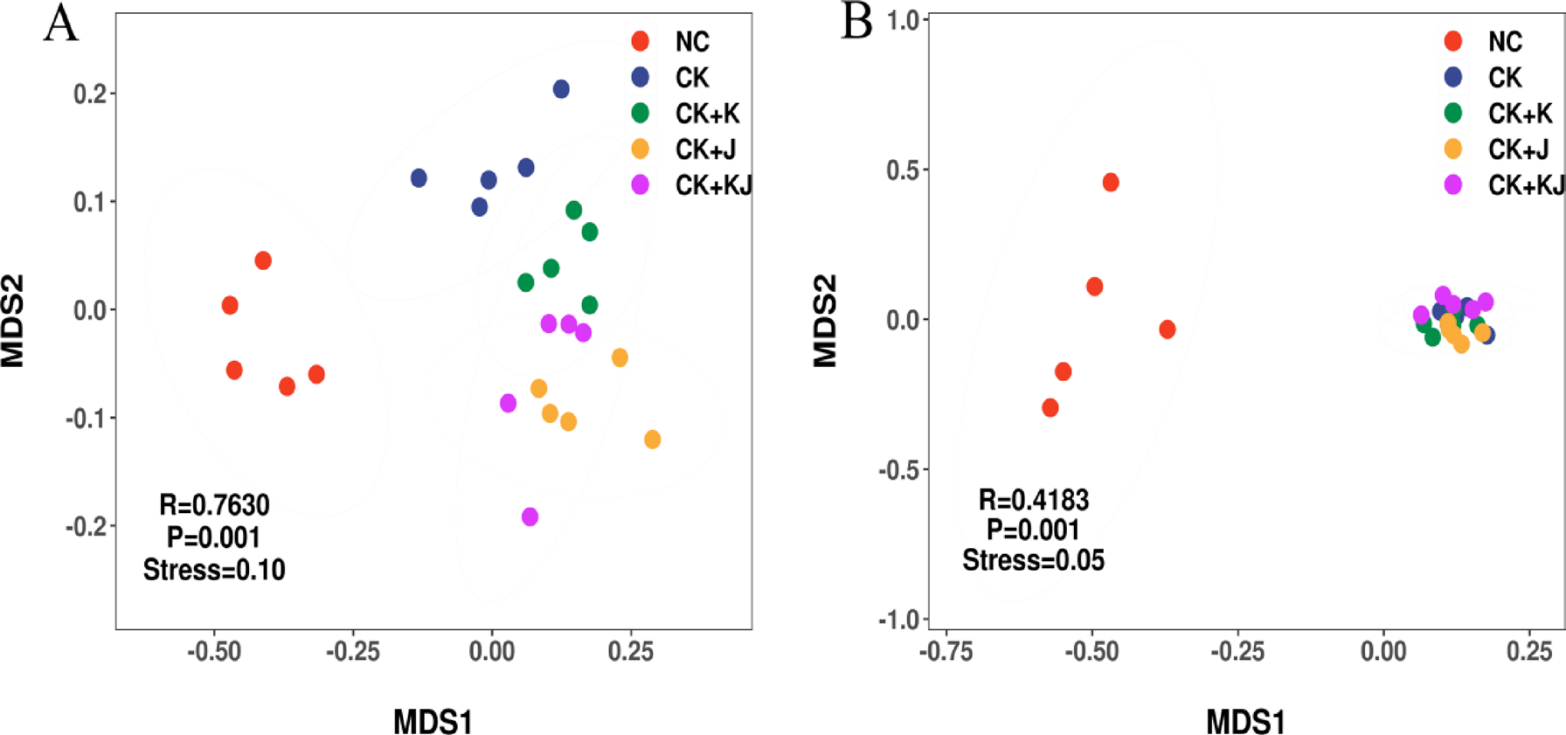
NMDS ordination based on Bray–Curtis distances for bacterial (**A**) and fungal (**B**) communities. NC, Noncontinuous cropping soil; CK, continuous cropping soil for 2 years as the control; CK + K, continuous cropping soil with *B. subtilis*; CK + J, continuous cropping soil with *B. mucilaginosus*; CK + KJ, continuous cropping soil with a combination of *B. subtilis* and *B. mucilaginosus*.

The NMDS analysis based on Bray–Curtis distance revealed distinct clustering patterns in microbial communities (Fig. 2). The CK treatment exhibited clear separation from NC treatment, indicating that continuous cropping effectively altered the composition of rhizosphere microbial communities (Fig. 2A and B). For bacterial communities, the three different *Bacillus* treatments formed distinct clusters in sugar beet rhizosphere soil, showing clear separation from CK. The CK + J treatment group exhibited the most significant differences, indicating that *Bacillus* application effectively altered the composition of rhizosphere bacterial communities (Fig. 2A). In contrast, fungal communities demonstrated varying patterns. The fungal communities in CK treatment clustered with those from various *Bacillus* treatments without clear separation, suggesting no significant impact of *Bacillus* treatment on fungal community composition. (Fig. 2B).

### Variations in microbial community structure

**Fig. 3:**
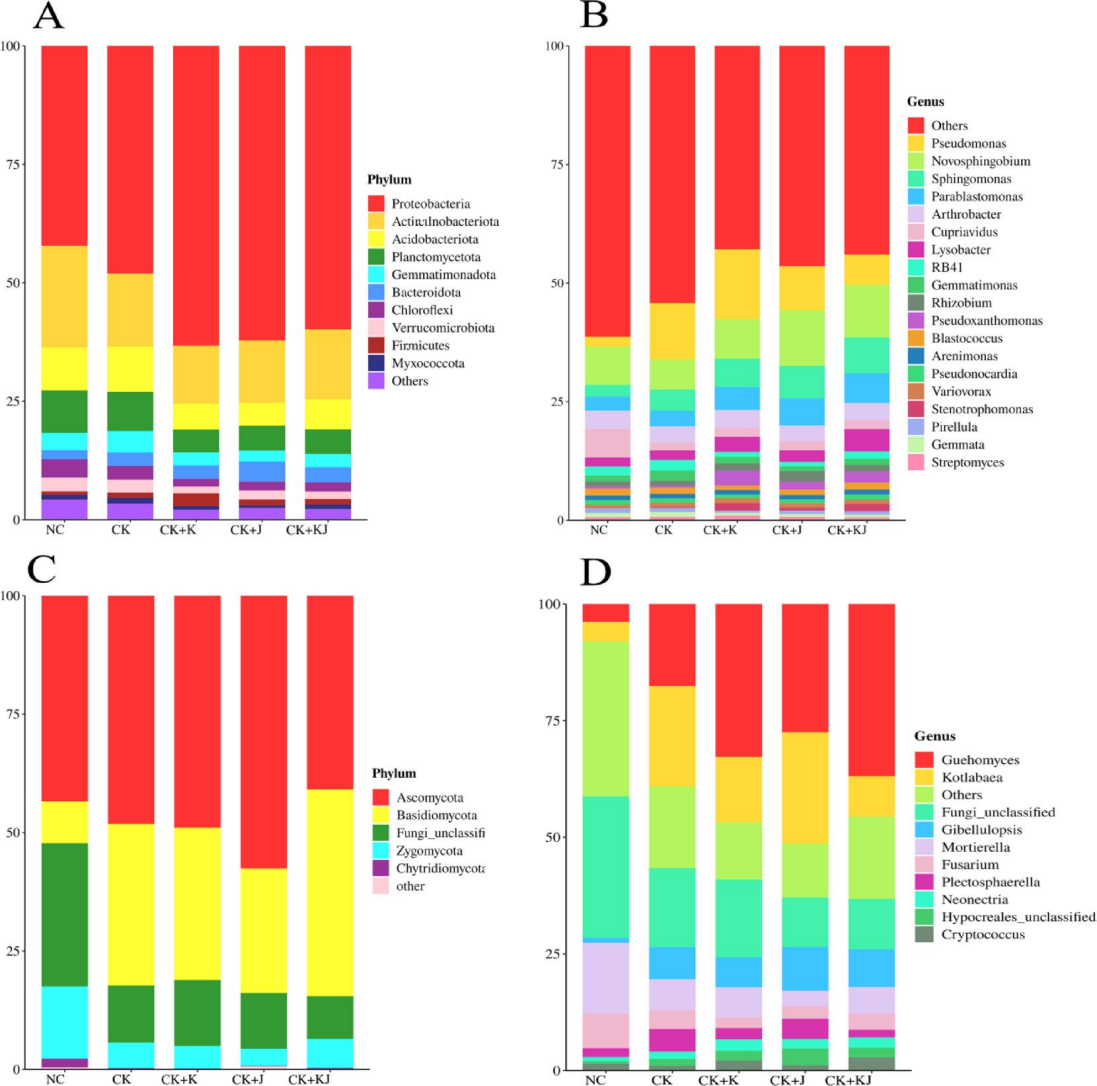
Relative abundance of bacterial and fungal phyla and genera in sugar beet rhizosphere soil under different treatments: (**A**) bacterial phyla, (**B**) bacterial genera, (**C**) fungal phyla, and (**D**) fungal genera. Different colors represent dominant taxa. NC, Noncontinuous cropping soil; CK, continuous cropping soil for 2 years as the control; CK + K, continuous cropping soil with *B. subtilis*; CK + J, continuous cropping soil with *B. mucilaginosus*; CK + KJ, continuous cropping soil with a combination of *B. subtilis* and *B. mucilaginosus*.

Bacterial community amplicon sequence variants (ASVs) were classified into 40 phyla, 143 classes, 325 orders, 537 families, 1152 genera, and 1913 species based on high-throughput sequencing results. Microbial community composition bar charts revealed that, after *Bacillus* treatment, the rhizosphere soil bacterial community of sugar beet differed significantly from that of CK and NC, while CK and NC also exhibited distinct compositions. Although the fungal communities showed no significant difference between *Bacillus* treatments and CK treatment, they demonstrated marked divergence from NC (Fig. 3). Proteobacteria, Acidobacteria, Actinobacteria, Planctomycetota, and Gemmatimonadota were the five dominant bacterial phyla across the five treatment groups. Proteobacteria of the five treatment groups exhibited the highest relative abundance, accounting for 42.16%, 48.67%, 63.24%, 62.18%, and 59.86% of bacterial communities, respectively. Compared with CK and NC, *Bacillus* treatment significantly increased Proteobacteria abundance in the rhizosphere soil while decreasing the abundance of Acidobacteria, Planctomycetota, and Gemmatimonadota (Fig. 3A). *Pseudomonas*, *Novosphingobium*, *Sphingomonas*, and *Parablastomonas* were the four dominant bacterial genera. *Pseudomonas* had the highest relative abundance in the CK + K treatment group, with *B. subtilis* inoculation further increasing its presence. In contrast, *Novosphingobium* exhibited higher relative abundance in the CK + J and CK + KJ groups compared with the CK and NC groups. Additionally, the relative abundance of *Sphingomonas* and *Parablastomonas* was higher in all three *Bacillus*-inoculated treatment groups compared with the CK and NC groups (Fig. 3B).

The high-throughput sequencing results revealed that fungal community ASVs from the five treatments were classified into 14 phyla, 46 classes, 96 orders, 195 families, 370 genera, and 635 species. Ascomycota, Basidiomycota, Zygomycota, and Chytridiomycota were the four dominant fungal phyla. The CK + J treatment increased Ascomycota abundance compared with the CK and NC. In contrast, the CK + KJ group exhibited the highest Basidiomycota abundance (Fig. 3C). *Guehomyces*, *Kotlabaea*, *Gibellulopsis*, and *Mortierella* were the primary fungal genera in sugar beet rhizosphere soil. The combined bacterial agent treatment (CK + KJ) significantly reduced the relative abundance of *Kotlabaea* in continuous cropping soils (CK), while maintaining levels comparable to that in NC (Fig. 3D).

### Differences in microbial community composition

**Fig. 4:**
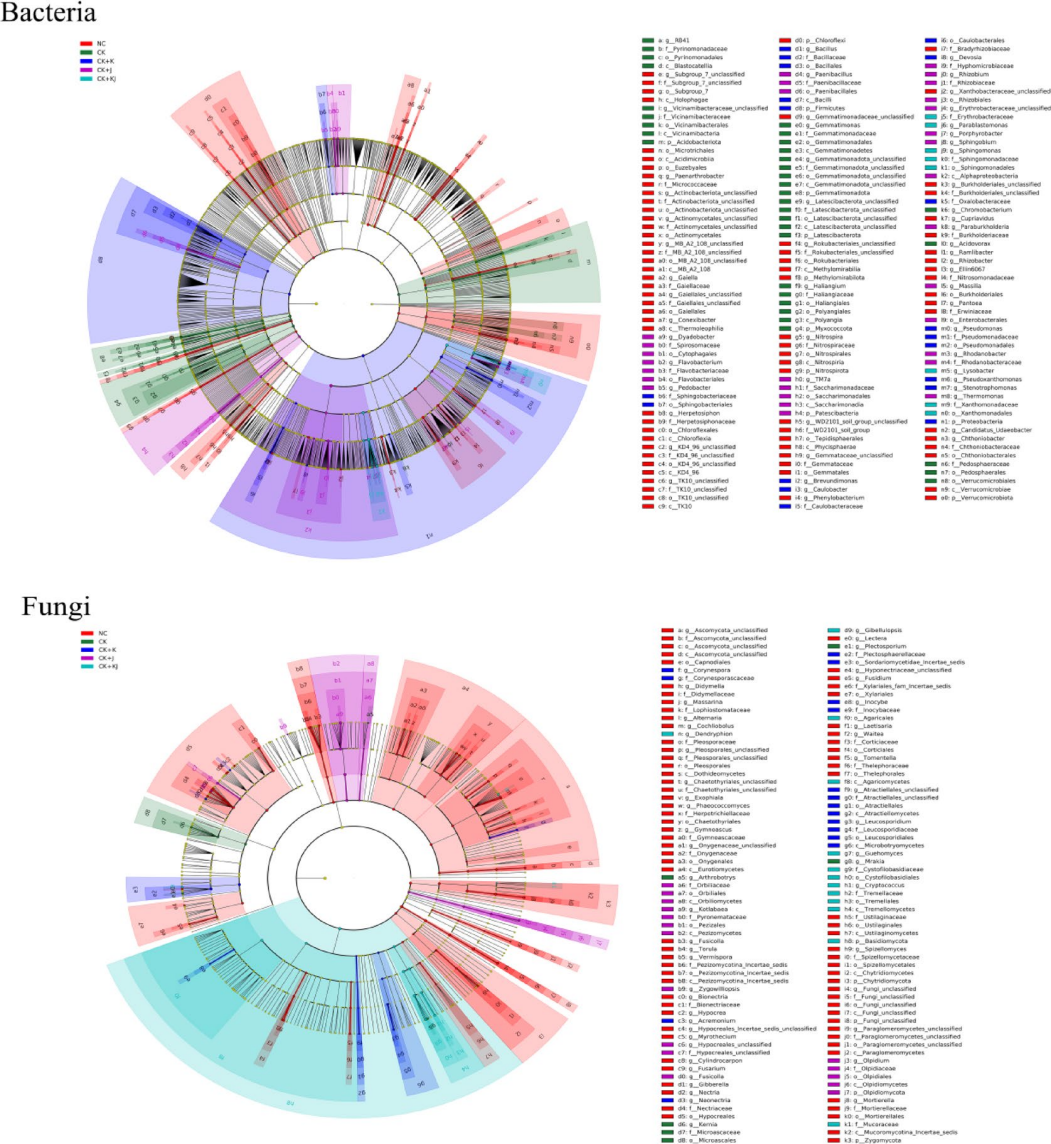
LEfSe-generated cladograms illustrating differences in bacterial and fungal abundance across taxonomic levels. Circular rings radiating outward represent phylum-to-genus classifications. Different color nodes in the branch diagram indicate microbial groups playing key biological roles in specific communities. The species names represented by English letters in the figure are listed in the legend on the right. NC, Noncontinuous cropping soil; CK, continuous cropping soil for 2 years as the control; CK + K, continuous cropping soil with *B. subtilis*; CK + J, continuous cropping soil with *B. mucilaginosus*; CK + KJ, continuous cropping soil with a combination of *B. subtilis* and *B. mucilaginosus*.

The differential responses of rhizosphere microbial communities in beetroot under various treatments were evaluated using LEfSe (LDA > 2) (Fig. 4). The cladogram analysis revealed a greater number of bacterial biomarker taxa compared with fungal counterparts. Except NC group, the CK + K group demonstrated extensive correlation nodes and occupied a larger area in the diagram, indicating that more microbial species played crucial biological roles following *B. subtilis* application.

Verrucomicrobiota, Acidobacteriota, Firmicutes, and Proteobacteria exhibited significant species differences in rhizosphere soil bacterial communities across the five treatment groups (Fig. 4, bacteria). At the phylum level, Chloroflexi and Verrucomicrobiota play key biological roles in NC bacterial communities. At the genus level, significant enrichment was observed in specific taxa including *Herpetosiphon*, *Gaiella*, *Conexibacter*, *Paenarthrobacter*, *Chthoniobacter*, and *Nitrospira*. Bacterial communities in continuous cropping soil played key biological roles at the phylum level, including, Acidobacteriota, Myxococcota, Latescibacterota, and Gemmatimonadota, with notable genus-level enrichment of *Gemmatimonas*, *Haliangium*, *RB41*, *Chromobacterium*, and *Acidovorax*. In CK + K treatment group, key biomarker taxa were in the following: *Bacillus* (order to genus), *Sphingomonas* (order to family), *Caulobacter* (order to genus), *Pseudoxanthomonas* (order to genus), and *Oxalobacteraceae* (family). The CK + J treatment group demonstrated a more complex biomarker profile, encompassing *Patescibacteria* (phylum to genus), *Flavobacterium* (order to genus), *Cytophagales* (order to genus), *Paenibacillus* (order to genus), *Enterobacterales* (order to genus), *Rhizobium* (order to genus), *Sphingobium* (genus), *Paraburkholderia* (genus), *Massilia* (genus), *Rhodanobacter* (family to genus), and *Thermomonas* (genus), all of which play important biological regulatory roles in rhizosphere soil. The CK + KJ treatment group resulted in the emergence of *Parablastomonas*, *Sphingomonas*, and *Lysobacter* as predominant genera. This multi-level taxonomic analysis underscored treatment-specific modulation of microbial consortia with distinct ecological implications.

In the rhizosphere soil fungal community, Basidiomycota was the dominant phylum, with some species showing significant differences across the five treatments (Fig. 4, Fungi). NC exhibited the highest diversity of biomarkers. At the phylum level, Chytridiomycota and Zygomycota were identified. At the genus level, the following taxa showed significant enrichment: *Paraglomeromycetes* (from class to genus), *Tomentella* (order to genus), *Ustilaginaceae* (order to genus), *Onygenaceae* (order to genus), *Chaetothyriales* (order to genus), *Pleosporales* (order to genus), as well as the genera *Phaeococcomyces*, *Exophiala*, *Gymnoascus*, *Hypocrea*, and *Nectria*. In CK treatment, *Mrakia* (genus), *Kernia* (genus), and *Arthrobotrys* (genus) were the dominant bacterial genera. *Atractiellomycetes* (class to genus), *Leucosporidiales* (order to genus), *Corynespora* (family and genus), and *Acremonium* (genus) played key biological roles in CK + K treatment. The CK + J treatment demonstrated significant enrichment of *Zygowilliopsis* (genus), *Fusicolla* (genus), and *Olpidium* (phylum to genus). The CK + KJ biomarkers included Basidiomycota at the phylum level and *Dendryphion*, *Guehomyces*, and *Gibellulopsis* at the genus level, all of which play crucial biological roles in the treated soil.

### Environmental factors influencing microbial community composition

**Fig. 5:**
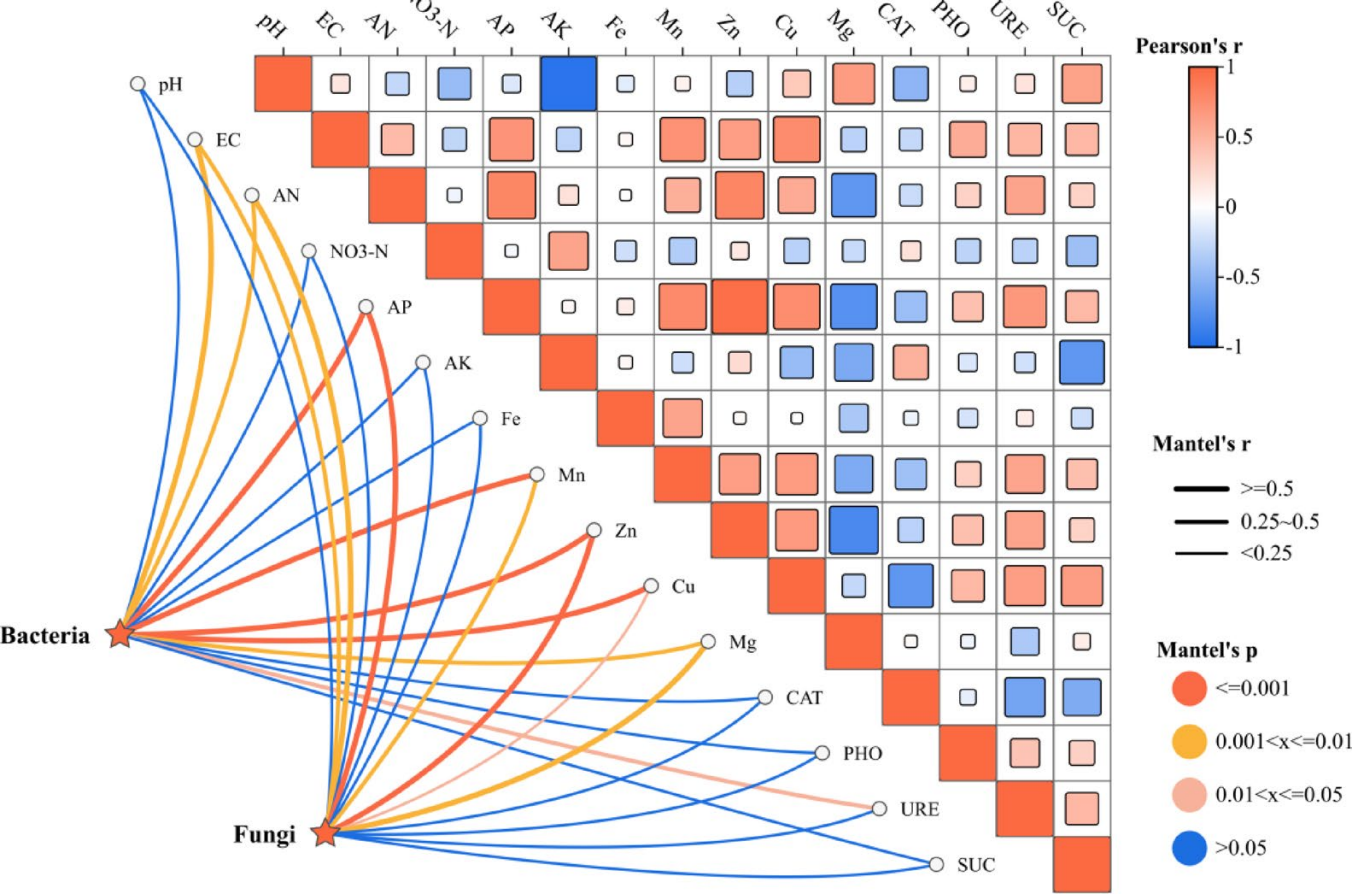
Environmental factors influencing soil microbial community composition in the sugar beet rhizosphere. Pearson correlations between microbial communities and environmental variables were analyzed using Mantel tests. Edge widths represent Mantel statistics, square sizes indicate correlation magnitudes, and colors correspond to Pearson coefficients. Line colors denote *P* values.

This study explored the environmental factors influencing microbial community composition by analyzing the correlation between rhizosphere soil microbial community composition and soil environmental factors (Fig. 5). The analysis revealed significant negative correlations between the following pairs: soil pH and available potassium (AK), AK and sucrase (SUC), as well as magnesium (Mg) and ammonium nitrogen (AN). In contrast, significant positive correlations were observed between AN and both available phosphorus (AP) and zinc (Zn), between AP and Zn, and between soil pH and SUC. The composition of the rhizosphere soil bacterial community exhibited significant correlations with electrical conductivity (EC), AN, AP, manganese (Mn), Zn, copper (Cu), Mg, and urease (URE) (*P* < 0.05), with the strongest correlations observed with AP, Mn, Zn and Cu. Meanwhile, fungal communities were also significantly correlated with EC, AN, AP, Mn, Zn, Cu, Mg and URE (*P* < 0.05), with the strongest correlations with AP and Zn. These results suggested that the diversity and composition of rhizosphere soil microbial communities were strongly influenced by environmental factors.

**Fig. 6:**
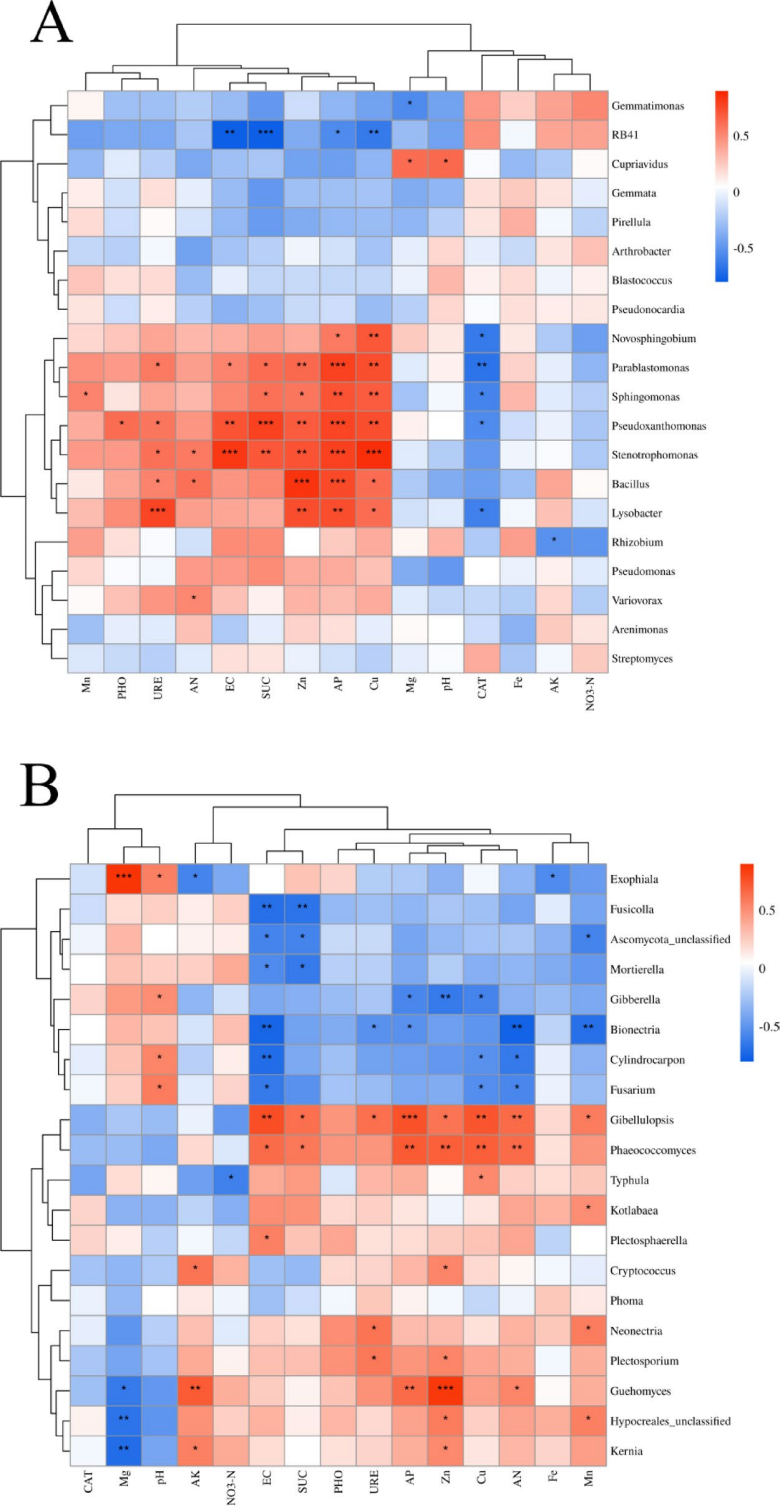
Clustering heatmap showing Spearman correlations between soil environmental factors and dominant bacterial genera (**A**) and fungal genera (**B**) (^*^0.01 < *P* ≤ 0.05, ^**^0.001 < *P* ≤ 0.01, ^***^P ≤ 0.001).

Spearman correlation coefficients between soil environmental factors and dominant microbial taxa were calculated and visualized using clustering heatmaps to highlight their relationships. The analysis showed that the number of bacterial and fungal genera significantly correlated with soil environmental factors, indicating that beetroot rhizosphere microbial communities were closely associated with and susceptible to soil environmental changes (Fig. 6). Further analysis revealed that Zn, AP and Cu were significantly positively correlated with the abundance of *Novosphingobium*, *Parablastomonas*, *Sphingomonas, Pseudoxanthomonas*, *Stenotrophomonas, Bacillus,* and *Lysobacter,* with AP exhibited an exceptionally strong positive correlation with *Parablastomonas*, *Stenotrophomonas*, *Pseudoxanthomonas*, and *Bacillus*, suggesting its significant promoting effect on the enrichment of these taxa. However, they showed a significant negative correlation with the abundance of *RB41*. Meanwhile, the abundance of *RB41* displayed a highly significantly negative correlation with EC and SUC, Whereas the abundance of *Pseudoxanthomonas* and *Stenotrophomonas* was significantly positively correlated with EC and SUC. Additionally, the abundance of *Lysobacter* was positively correlated with URE. Different soil enzyme activities differentially affected soil bacterial communities: CAT, URE, and SUC had more pronounced effects on bacterial communities compared with PHO. SUC and URE exhibited significant positive correlations with *Parablastomonas, Pseudoxanthomonas* and *Lysobacter*, whereas CAT showed marked negative correlations with those bacterial genera (Fig. 6A).

The analysis of soil environmental factors influencing rhizosphere fungal communities showed that *Gibellulopsis, Phaeococcomyces* and *Guehomyces* demonstrated significant positive correlations with most factors, such as EC, SUC, AP, Zn, AP, Cu, AN and Mn, whereas *Gibberella, Bionectria, Cylindrocarpon* and *Fusarium* exhibited significant negative correlations with those factors. Additionally, Mg not only showed a highly significant positive correlation with *Exophiala* but also significant negative correlations with *Kernia*, *Hypocreales*, and *Guehomyces* (Fig. 6B). In summary, environmental factors and microbial communities are closely interconnected, with changes in one component likely inducing alterations in the other.

### Prediction of microbial community function

**Fig. 7:**
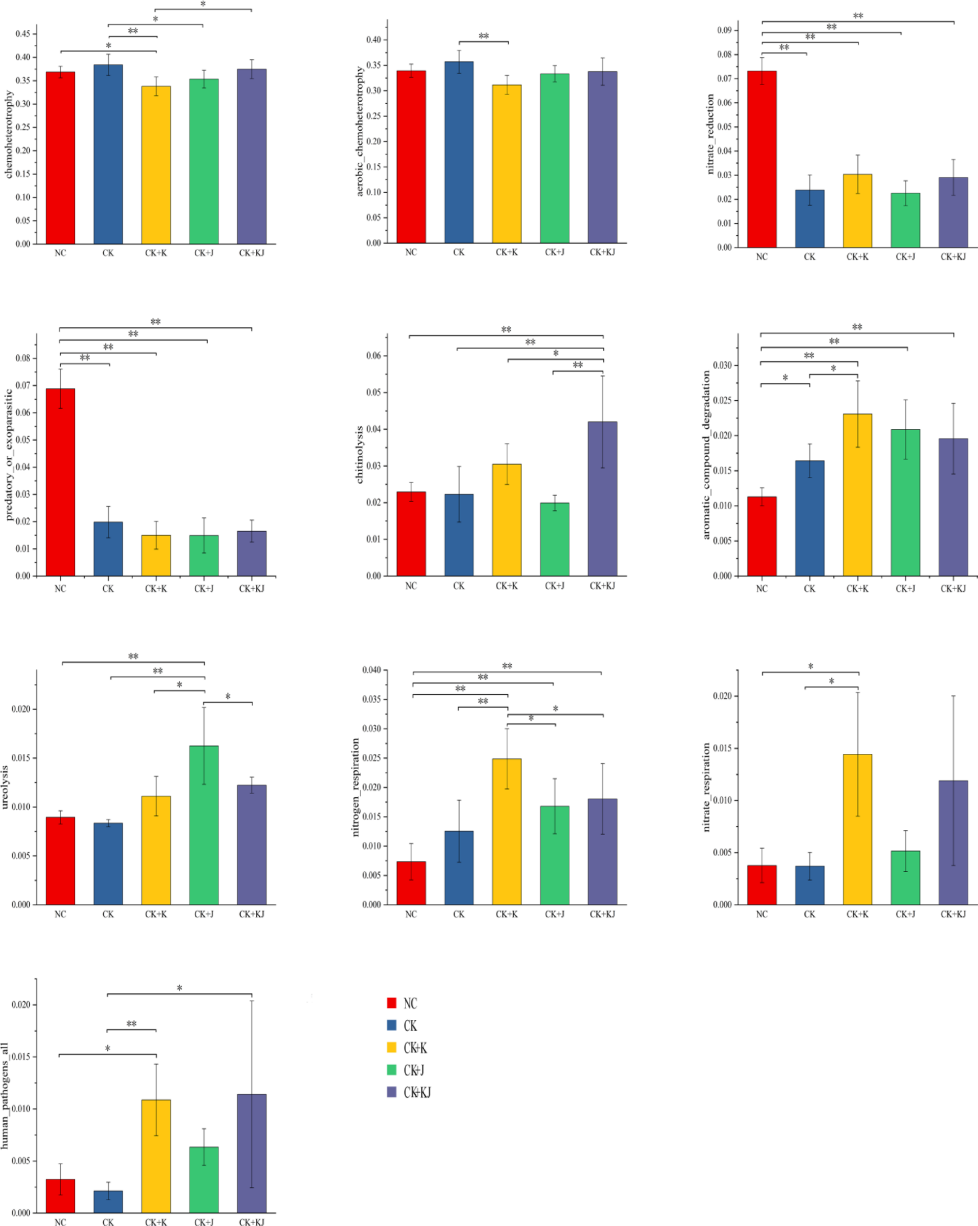
Bacterial ecological function enrichment under different treatments "^*^" : *P* ≤ 0.05; “^**^” : *P* ≤ 0.01. NC, Noncontinuous cropping soil; CK, continuous cropping soil for 2 years as the control; CK + K, continuous cropping soil with *B. subtilis*; CK + J, continuous cropping soil with *B. mucilaginosus*; CK + KJ, continuous cropping soil with a combination of *B. subtilis* and *B. mucilaginosus*.

Based on the FAPROTAX database (v1.2.1, http://www.loucalab.com/archive/FAPROTAX/lib/php/index.php?section=Download), this study analyzed the rhizosphere soil bacterial communities under different treatments and predicted 90 potential ecological functions. The variation characteristics of the top 10 bacterial functional groups in terms of average relative abundance across samples are shown in Fig. 7. Chemoheterotrophy and aerobic chemoheterotrophy were identified as the predominant bacterial ecological functions across all treatment groups, collectively accounting for half of all predicted functions. Other relatively abundant functional categories included nitrate reduction, predatory or exoparasitic behavior, chitinolysis, and aromatic compound degradation.

Differential analysis of functional categories among treatment groups (Fig. 7) revealed that the CK + K and CK + J treatments significantly reduced the relative abundance of chemoheterotrophy compared with the CK group, whereas CK + KJ showed no significant difference in chemoheterotroph abundance relative to the CK or NC, suggesting that the co-application of *B. subtilis* and *B. mucilaginosus* neutralized their individual inhibitory effects. Meanwhile, CK + K treatment also significantly reduced the relative abundance of aerobic chemoheterotrophy compared with that in the CK group. The NC group demonstrated significantly higher abundance of nitrate reduction processes and predatory or exoparasitic functions compared to all other treatment groups (CK, CK + K, CK + J, and CK + KJ), whereas no significant differences in these functional traits were detected among the CK, CK + K, CK + J, and CK + KJ groups. The CK + KJ treatment showed the highest chitinolytic activity, with significant differences compared with other groups, followed by the CK + K treatment. Regarding aromatic compound degradation and nitrogen respiration functions, all three bacterial inoculation treatments outperformed the NC group, with CK + K treatment demonstrated the most pronounced effect, showed significantly greater than both the NC group and CK group. In terms of ureolysis function, the CK + J treatment showed the highest abundance and significant differences compared with all other groups. For nitrate respiration, the CK + K treatment displayed the highest abundance, with significantly higher than that in the NC group and CK group, followed by the CK + KJ treatment, whereas no significant difference was observed between NC and CK controls. The human pathogens (all) function significantly increased under both the CK + K and CK + KJ treatments compared with controls. These findings indicated that the two *Bacillus* agents and their mixture exerted minimal effects on the relative abundance of chemoheterotrophy and aerobic chemoheterotrophy functions in rhizosphere soil bacterial communities, but significantly modulated several less dominant functional types.

**Fig. 8:**
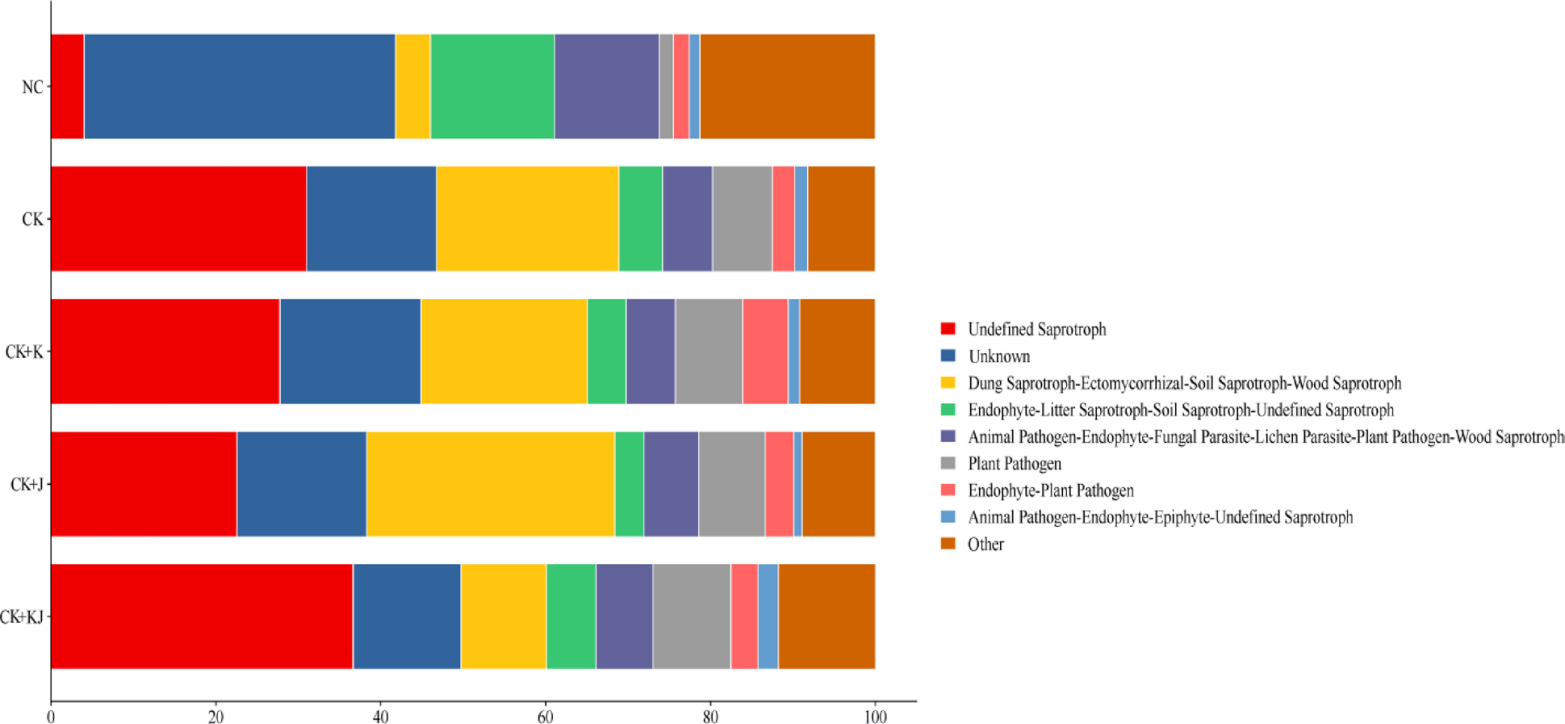
Distribution and abundance of fungal trophic modes under different treatments. NC, Noncontinuous cropping soil; CK, continuous cropping soil for 2 years as the control; CK + K, continuous cropping soil with *B. subtilis*; CK + J, continuous cropping soil with *B. mucilaginosus*; CK + KJ, continuous cropping soil with a combination of *B. subtilis* and *B. mucilaginosus*.

The functional prediction of fungal communities in sugar beet rhizosphere soils under different treatments was performed using the FunGuild tool (v1.0, http://stbates.org/funguild_db.php). The analysis revealed distinct patterns in the relative abundance and distribution of fungal trophic modes across treatments, as illustrated in Fig. 8. Except for the NC treatment group, the three most abundant fungal trophic types under other treatments were Undefined Saprotroph (24.07–36.65%), Unknown trophic mode (13.07–17.69%), and Dung Saprotroph–Ectomycorrhizal–Soil Saprotroph–Wood Saprotroph (10.36–34.61%). The CK + KJ treatment exhibited the highest relative abundance of Undefined Saprotroph. The CK + J treatment showed a significant difference in Dung Saprotroph–Ectomycorrhizal–Soil Saprotroph–Wood Saprotroph abundance compared with the CK. The relative abundance of Unknown trophic mode (37.77%), Endophyte–Litter Saprotroph–Soil Saprotroph–Undefined Saprotroph (15.05%), and Animal Pathogen–Endophyte–Fungal Parasite–Lichen Parasite–Plant Pathogen–Wood Saprotroph (12.79%) were significantly higher under the NC treatment than under other treatments. Furthermore, NC displayed the lowest relative abundance of Plant Pathogen (1.73%) and Endophyte–Plant Pathogen (1.89%). No significant differences were observed among treatments for Animal Pathogen–Endophyte–Epiphyte–Undefined Saprotroph. These findings demonstrated that the CK + J treatment significantly influenced the abundance of Dung Saprotroph–Ectomycorrhizal–Soil Saprotroph–Wood Saprotroph in fungal communities, while showing minimal effects on other trophic types. Comparatively, the NC exhibited marked differences in fungal community composition compared with other treatments.

### Correlations between microbial community and plant growth

**Fig. 9:**
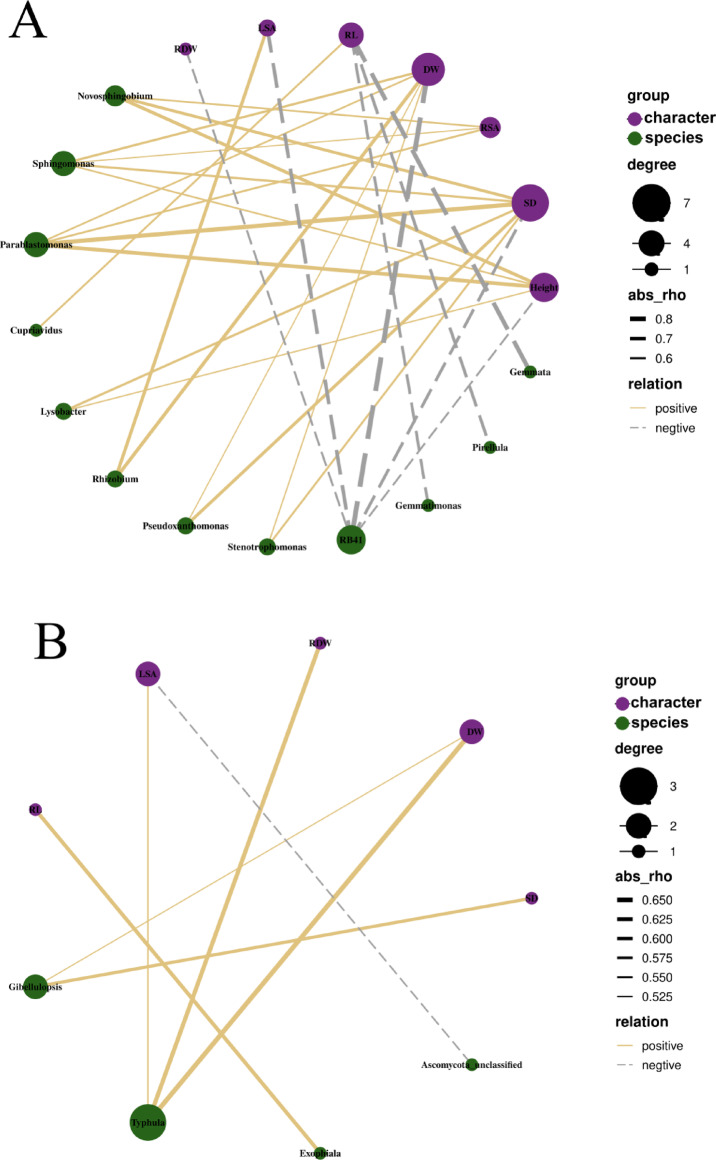
The correlation network diagram between the composition of microbial communities and plant growth traits. (**A**) Represents the correlation between soil bacteria and plant growth traits, and (**B**) represents the correlation between soil fungi and plant growth traits. Nodes represent the types of soil microorganisms and plant growth traits. The yellow solid line indicates a positive correlation, while the gray dotted line indicates a negative correlation. The thickness of the connection lines and the size of the nodes respectively reflect the intensity of the association between species and traits and the species abundance (or association degree).

The correlation network shows that most bacterial genera are positively correlated with plant growth traits(Fig. 9A). Among these traits, aboveground dry weight(DW), stem diameter(SD), and plant height(Height) were strongly correlated with most bacterial genera. Three genera(*Novosphingobium*, *Sphingobium*, and *Parablastomonasdis*) exhibited positive correlations with most plant growth traits, notably with root surface area(RSA), Height, and SD. Meanwhile, *Rhizobium*, *Pseudoxanthomonas* and *Stenotrophomonas* are positively correlated with DW, SD and leaf surface area (LSA). In contrast, the bacterial taxa *RB41*, *Gemmatimonas*, *Pirellula*, and *Gemmata* were negatively correlated with plant growth traits. Specifically, whereas *Gemmatimonas*, *Pirellula*, and *Gemmata* showed negative correlations with root length (RL), *RB41* demonstrates broad negative associations with most traits, particularly with DW. Correlations between fungal communities and plant growth were considerably weaker than bacterial ones (Fig. 9B). DW, RDW, and LSA all demonstrated significant positive correlations with *Typhula*, while SD and RL showed positive correlations specifically with *Gibellulopsis* and *Exophiala*, respectively. In contrast, *Ascomycota_unclassified* displays negative correlations with LSA.

## Discussion

As one of the most effective biocontrol agents, *Bacillus* species promote plant growth, regulate soil microbial communities, and exhibit strong antagonistic effects against certain pathogens, demonstrating significant potential in alleviating continuous cropping obstacles^34,44^. Additionally, prior investigations have demonstrated the ability of *Bacillus* to colonize the rhizosphere soil^45,46^. This study investigated the effects of *B. subtilis* and *B. mucilaginosus* inoculants on the growth and development of continuously cropped sugar beet seedlings and their impact on rhizosphere soil physicochemical properties and microbial communities.

Compared with the continuous cropping CK, sugar beet seedlings treated with *B. subtilis*, *B. mucilaginosus*, or a combination of both showed significant improvements in growth parameters, including stem diameter (SD), plant height(Height), and root dry weight (RDW). The level of beet seedlings could recover to the noncontinuous cropping group (NC) level or even higher than the NC group index. These findings aligned with previous studies on crops such as tomato and radish^47–49^. Continuous cropping induced soil acidification, characterized by decreased pH and altered physicochemical properties^37^. This study revealed that *Bacillus* inoculants significantly increased soil pH, available phosphorus (AP) content, and urease (URE) and sucrase (SUC) activities while reducing available potassium (AK) content and Catalase (CAT) activity. These trends in environmental factor changes were consistent with previous studies^48,50,51^. The results demonstrated that both *Bacillus* species significantly promoted sugar beet seedling growth and regulated soil physicochemical properties.

*Bacillus* species influence plant growth by regulating rhizosphere microbial community structure and diversity^27,29^. High-throughput sequencing was performed on rhizosphere soil from continuous cropping sugar beet seedlings and *Bacillus*-treated groups. Compared to both the CK and NC groups, bacterial community abundance significantly decreased following treatment with either *Bacillus* inoculant or their combination. At the same time, the single use of the two *Bacillus* also reduced the abundance of fungal communities compared to the NC group, consistent with the findings of Sui et al^34^. However, *Bacillus* treatments did not significantly impact fungal and bacterial diversity. NMDS results further indicated that *Bacillus* treatments altered bacterial community composition in continuous cropping rhizosphere soil but did not affect fungal community structure.

The analysis of microbial community composition revealed that Proteobacteria, Acidobacteria, and Actinobacteria were the dominant bacterial phyla in sugar beet rhizosphere soil. Among these, Proteobacteria had the highest relative abundance, making it the dominant phylum, consistent with findings of Cui, Shen, and others^9,50^. *Bacillus* treatments significantly increased Proteobacteria abundance compared with CK and NC. *Pseudomonas*, *Novosphingobium*, and *Sphingomonas* within the phylum Proteobacteria were the most abundant genera, with higher relative abundance in inoculant-treated groups than in the CK group. *Pseudomonas* and *Sphingomonas* species, recognized as beneficial rhizobacteria, have been shown to promote plant growth under stress conditions by producing volatile compounds and secondary metabolites or by modulating plant hormone balance and signal transduction^52–54^. The fungal communities in rhizosphere soil were predominantly composed of Ascomycota and Basidiomycota, aligning with previous findings on potato and watermelon soils^55,56^. The influence of *Bacillus* inoculants on fungal community structure was less pronounced than their impact on bacterial communities. Applying microbial agents might decrease the relative abundance of certain fungal phyla. For instance, Ascomycota was dominant in both control and *Bacillus*-treated groups; however, its relative abundance was reduced under combined *Bacillus* inoculant treatment compared with other treatments. The relative abundance of *Kotlabaea* within Ascomycota also showed a significant reduction, consistent with previous findings^50^. LEfSe analysis further revealed that bacterial taxa exhibited more biomarker species than fungi, with additional bacterial groups playing crucial biological roles following *Bacillus* treatment. These results indicated that *Bacillus* inoculants improved the rhizosphere microecological environment and promoted beneficial bacterial enrichment.

Soil microbial communities serve as integrated responses to environmental factors and plant interactions, effectively reflecting the trends in environment–plant dynamics. The analysis revealed that most environmental factors correlated with bacterial communities also exhibited significant associations with fungal communities, demonstrating that specific soil parameters (e.g., AP and zinc(Zn)) were more closely linked to rhizosphere soil microbial communities. Consequently, the responses of microbial communities to environmental factors may influence microbial abundance and composition, ultimately affecting host plants. The correlation analysis between microbial communities and environmental factors identified that AP, Zn, and copper(Cu) exhibited significant positive correlations with the majority of bacterial and fungal genera and significantly promoted the enrichment of these microbial taxa. These findings suggested that partial environmental factors might regulate microbial diversity while indirectly shaping soil microbial community composition.

The functional prediction results of the beet rhizosphere microecology indicated that in terms of bacterial functional prediction, the addition of *Bacillus* inoculants had minimal impact on the relative abundance of the primary environmental functions within the bacterial community. However, it exerted a regulatory effect on certain less dominant functional types. Specifically, the nitrate respiration, nitrogen respiration, and chitinolytic functions of the bacterial community were significantly enhanced by the inoculation. Regarding fungal functional prediction, fungi in the rhizosphere soil were predominantly saprotrophic, consistent with the trophic modes observed for fungi in other soil types^57^. The abundance of Undefined Saprotroph fungi reached its maximum when the two *Bacillus* strains were applied in combination. Furthermore, the relative abundance of the Dung Saprotroph-Ectomycorrhizal-Soil Saprotroph-Wood Saprotroph functional guild increased substantially following the addition of *B. mucilaginosus*. This indicates that the bacterial inoculants elevated the abundance of nutritionally specialized saprotrophic fungi in the soil, which are potentially involved in modulating the structure of soil carbon and nitrogen components.

Soil microbial community composition is closely linked to plant growth performance. Our results indicate that a majority of microbial genera show significant positive correlations with key plant growth traits, with bacteria contributing more significantly than fungi. This implies that specific bacterial genera in the soil may play pivotal roles in influencing the expression of these traits. Notable examples include *Novosphingobium*, *Sphingobium*, and *Parablastomonas.*

## Conclusions

Under continuous cropping conditions, the application of *B. subtilis* and *B. mucilaginosus* significantly promoted sugar beet growth, increased soil pH while reducing acidity, enhanced the synthesis of various soil nutrients, improved URE and SUC activities, and modified soil microbial community structure. Introducing the two aforementioned *Bacillus* species increased the relative abundance of Proteobacteria while decreasing Ascomycota abundance in rhizosphere soil, thereby optimizing microbial community composition and facilitating the enrichment of beneficial microorganisms such as *Pseudomonas*, *Novosphingobium*, and *Sphingomonas*. And most of which are positively correlated with plant growth, constitute a key group of beneficial taxa. Furthermore, the addition of *Bacillus* inoculants enriched a substantial number of functional biomarkers associated with potassium solubilization, nitrogen fixation, salt and drought tolerance, and plant growth promotion. Concurrently, it enhanced the nitrate respiration, nitrogen respiration, and chitinolytic functions within the bacterial community. Additionally, the inoculants elevated the abundance of nutritionally specialized saprotrophic fungi in the soil, thereby contributing to the cycling of soil carbon and nitrogen. This study provides an effective biological strategy for alleviating continuous cropping obstacles in sugar beet production, demonstrating that *B. subtilis* and *B. mucilaginosus* serve as efficient bioagents to mitigate the adverse effects of monoculture. These findings advance the understanding of plant–microbiota interactions in continuous cropping systems and offer critical insights for future applications of microbial inoculants in addressing replanting challenges.

Finally, it is important to acknowledge the limitations inherent in this pot-based study. Although pot experiments facilitate variable control, the ecological conditions differ from those in field environments, thus the generalizability of the findings to field applications requires further validation.

## Data Availability

The datasets generated during and/or analyzed during the current study are available in the NCBI Sequence Read Archive (SRA) database under accession numbers PRJNA1310802 (ITS) and PRJNA1310801 (16S).

## References

[1] Steven, Z., Zhang, R. H. & Stephen, K. In *In integrated processing technologies for food and agricultural by-products* (ed. Stephen, Z.) 331–351 (Academic Press, Cambridge, 2019).

[2] Chhikara, N. et al. Bioactive compounds of beetroot and utilization in food processing industry: A critical review. *Food Chem.* **272**, 192–200. 10.1016/j.foodchem.2018.08.022 (2018). 30309532 10.1016/j.foodchem.2018.08.022

[3] Mall, A. K. et al. Sugar beet cultivation in India: Prospects for bio-ethanol production and value-added co-products. *Sugar Tech.* **23**(6), 1218–1234. 10.1007/s12355-021-01007-0 (2021). 34248307 10.1007/s12355-021-01007-0 PMC8261398

[4] Jbawi, E. et al. Genotype: Environment interaction study in sugar beet (*Beta**vulgaris* L.). *Int. J. Environ.* **5**(3), 74–86. 10.3126/ije.v5i3.15706 (2016).

[5] Holmquist, L. et al. Major latex protein-like encoding genes contribute to *Rhizoctonia**solani* defense responses in sugar beet. *Mol. Genet. Genom.* **296**(1), 155–164. 10.1007/s00438-020-01735-0 (2020). 10.1007/s00438-020-01735-0 PMC7840631 33118051

[6] Wu, X. et al. The effect of long-term continuous cropping of black pepper on soil bacterial communities as determined by 454 pyrosequencing. *PLoS ONE* **10**(8), e0136946–e0136946. 10.1371/journal.pone.0136946 (2015). 26317364 10.1371/journal.pone.0136946 PMC4552827

[7] Zhu, B. et al. Diversity of rhizosphere and endophytic fungi in *Atractylodes**macrocephala* during continuous cropping. *PeerJ* **8**, e8905. 10.7717/peerj.8905 (2020). 32292655 10.7717/peerj.8905 PMC7144587

[8] Tang, J., Xue, Z. Q., Daroch, M. & Ma, J. Impact of continuous *Salvia**miltiorrhiza* cropping onrhizosphere actinomycetes and fungi communities. *Ann. Microbiol.* **65**(3), 1267–1275. 10.1007/s13213-014-0964-2 (2015).

[9] Cui, R. F. et al. The response of sugar beet rhizosphere micro-ecological environment to continuous cropping. *Front. Microbiol.* **13**, 956785. 10.3389/fmicb.2022.956785 (2022). 36160206 10.3389/fmicb.2022.956785 PMC9490479

[10] Kui, L. et al. Large-scale characterization of the soil microbiome in ancient tea plantations using high-throughput 16S rRNA and internal transcribed spacer amplicon sequencing. *Front. Microbiol.* **12**, 745225. 10.3389/fmicb.2021.745225 (2021). 34721345 10.3389/fmicb.2021.745225 PMC8555698

[11] Pimentel, D. et al. Conserving biological diversity in agricultural/forestry systems. *Bioscience* **42**(5), 354–362. 10.2307/1311782 (1992).

[12] Tkacz, A. et al. Stability and succession of the rhizosphere microbiota depends upon plant type and soil composition. *ISME J.* **9**, 2349–2359 (2015). 25909975 10.1038/ismej.2015.41 PMC4611498

[13] Tong, J., Cao, M. & Wang, R. Effects of restoration time on microbial diversity in rhizosphere and non-rhizosphere soil of *Bothriochloa**ischaemum*. *Int. J. Environ. Res. Public Health.* **15**, 2155. 10.3390/ijerph15102155 (2018). 30274384 10.3390/ijerph15102155 PMC6210566

[14] Zheng, X. et al. Effects of a microbial restoration substrate on plant growth and rhizo-sphere bacterial community in a continuous tomato crop-ping greenhouse. *Sci. Rep.* **10**, 13729. 10.1038/s41598-020-70737-0 (2020). 32792530 10.1038/s41598-020-70737-0 PMC7426824

[15] Cao, B. et al. Insight into the variation of bacterial structure in atrazine-contaminated soil regulating by potential phytoremediator: *Pennisetum**americanum* (L.) K. Schum.. *Front Microbiol.* **9**, 864. 10.3389/fmicb.2018.00864 (2018). 29780374 10.3389/fmicb.2018.00864 PMC5945882

[16] Das, S. et al. Taxonomic and functional responses of soil microbial communities to slag-based fertilizer amendment in rice cropping systems. *Environ. Int.* **127**, 531–539. 10.1016/j.envint.2019.04.012 (2019). 30981911 10.1016/j.envint.2019.04.012

[17] Kang, P. et al. A comparison of microbial composition under three tree ecosystems using the stochastic process and network complexity approaches. *Front. Microbiol.* **13**, 1018077. 10.3389/fmicb.2022.1018077 (2022). 36299726 10.3389/fmicb.2022.1018077 PMC9589112

[18] Zhang, X. et al. Plant growth and development of tropical seagrass deter-mined rhizodeposition and its related microbial community. *Mar Pollut Bull.* **199**, 115940. 10.1016/j.marpolbul (2024). 38150979 10.1016/j.marpolbul.2023.115940

[19] Liu, J. et al. Development of a soil quality index for *Camellia**oleifera* forestland yield under three different parent materials in Southern China. *Soil Till Res.* **176**, 45–50. 10.1016/j.still.2017.09.013 (2018).

[20] Qi, R. M. et al. Temperature effects on soil organic carbon, soil labile organic carbon fractions, and soil enzyme activities under long-term fertilization regimes. *Appl. Soil Ecol.* **102**, 36–45. 10.1016/j.apsoil.2016.02.004 (2016).

[21] Ahsan, T. et al. Effects of microbial agent and microbial fertilizer input on soil microbial community structure and diversity in a peanut continuous cropping system. *J. Adv. Res.* **64**, 1–13. 10.1016/j.jare.2023.11.028 (2024). 38030126 10.1016/j.jare.2023.11.028 PMC11464484

[22] Li, M. et al. Effects of continuous cropping of sugar beet (*Beta**vulgaris* L.) on its endophytic and soil bacterial community by high-throughput sequencing. *Ann. Microbiol.* **70**, 39. 10.1186/s13213-020-01583-8 (2020).

[23] Sun, R. C. et al. Bacterial diversity in soils subjected to long-term chemical fertilization can be more stably maintained with the addition of livestock manure than wheat straw. *Soil Biol. Biochem.* **88**, 9–18. 10.1016/j.soilbio.2015.05.007 (2015).

[24] Zhang, S. N. et al. Cow manure application effectively regulates the soil bacterial community in tea plantation. *BMC Microbiol.* **20**(1), 190. 10.1186/s12866-020-01871-y (2020). 32611380 10.1186/s12866-020-01871-y PMC7329415

[25] Cai, X. Y. et al. Effects and mechanisms of symbiotic microbial combination agents to control tomato *fusarium* crown and root rot disease. *Front. Microbiol.* **12**, 629793. 10.3389/fmicb.2021.629793 (2021). 34220730 10.3389/fmicb.2021.629793 PMC8245789

[26] Duan, Y. N. et al. The phlorizin-degrading *Bacillus**licheniformis* XNRB-3 mediates soil microorganisms to alleviate apple replant disease. *Front. Microbiol.* **13**, 839484. 10.3389/fmicb.2022.839484 (2022). 35308362 10.3389/fmicb.2022.839484 PMC8927668

[27] Olanrewaju, O. S., Ayilara, M. S., Ayangbenro, A. S. & Babalola, O. O. Genome mining of three plant growth-promoting *bacillus* species from maize rhizosphere. *Appl. Biochem. Biotechnol.* **193**(12), 3949–3969. 10.1007/s12010-021-03660-3 (2021). 34529229 10.1007/s12010-021-03660-3 PMC8610958

[28] Solanki, M. K. et al. Diversity and antagonistic potential of Bacillusspp. associated to the rhizosphere of tomato for the management of *Rhizoctonia**solani*. *Biocontrol. Sci. Technol.* **22**(2), 203–217. 10.1080/09583157.2011.649713 (2012).

[29] Chen, X. R. et al. *Bacillus**velezensis* strain GUMT319 reshapes soil microbiome biodiversity and increases grape yields. *Biology.* **11**(10), 1486–1486. 10.3390/biology11101486 (2022). 36290389 10.3390/biology11101486 PMC9598471

[30] Shao, F. F., Tao, W. H., Yan, H. K. & Wang, Q. J. Effects of microbial organic fertilizer (MOF) application on desert soil enzyme activity and jujube yield and quality. *Agronomy* **13**(9), 2427–2427. 10.3390/agronomy13092427 (2023).

[31] Gajbhiye, A., Rai, A. R. & Meshram, S. U. Isolation, evaluation and characterization of *Bacillus**subtilis* from cotton rhizospheric soil with biocontrol activity against *Fusarium**oxysporum*. *World. J. Microb. Biot.* **26**(7), 1187–1194. 10.1007/s11274-009-0287-9 (2009). 10.1007/s11274-009-0287-9 24026922

[32] Govindasamy, V. et al. *Bacillus* and *Paenibacillus* spp.: Potential PGPR for sustainable agriculture. 18. 10.1007/978-3-642-13612-2_15 (2010).

[33] Qiao, J. Q. et al. Addition of plant-growth-promoting *Bacillus**subtilis* PTS-394 on tomato rhizosphere has no durable impact on composition of root microbiome. *BMC Microbiol.* **17**(1), 131. 10.1186/s12866-017-1039-x (2017). 28583081 10.1186/s12866-017-1039-x PMC5460418

[34] Sui, J. K. et al. Effects of *Bacillus**subtilis* T6–1 on the rhizosphere microbial community structure of continuous cropping poplar. *Biology.* **11**(5), 791–791. 10.3390/biology11050791 (2022). 35625519 10.3390/biology11050791 PMC9138279

[35] Wang, D. et al. Genomic insights and functional analysis reveal plant growth promotion traits of *Paenibacillus**mucilaginosus* G78. *Genes* **14**(2), 392–392. 10.3390/genes14020392 (2023). 36833318 10.3390/genes14020392 PMC9956331

[36] Lu, R. K. Soil and agricultural chemistry analysis (ed. Lu, R. K.) pp. 248–254 (1999).

[37] Huang, W. J. et al. Effects of continuous sugar beet cropping on rhizospheric microbial communities. *Genes* **11**(1), 13–13. 10.3390/genes11010013 (2019). 31877827 10.3390/genes11010013 PMC7017100

[38] Kõljalg, U. et al. The taxon hypothesis paradigm: On the unambiguous detection and communication of taxa. *Microorganisms.* **8**(12), 1910–1910. 10.3390/microorganisms8121910 (2020). 33266327 10.3390/microorganisms8121910 PMC7760934

[39] Quast, C. et al. The SILVA ribosomal RNA gene database project: Improved data processing and web-based tools. *Nucleic Acids Res.* **41**(D1), D590–D596. 10.1093/nar/gks1219 (2012). 23193283 10.1093/nar/gks1219 PMC3531112

[40] Li, Q., Huang, Y., Xin, S. & Li, Z. Y. Comparative analysis of bacterioplankton assemblages from two subtropical karst reservoirs of southwestern China with contrasting trophic status. *Sci. Rep.* **10**(1), 22296. 10.1038/s41598-020-78459-z (2020). 33339847 10.1038/s41598-020-78459-z PMC7749139

[41] Schloss, P. D. et al. Introducing mothur: Open-source, platform-independent, community-supported software for describing and comparing microbial communities. *Appl. Environ. Microbiol.* **75**(23), 7537–7541. 10.1128/AEM.01541-09 (2009). 19801464 10.1128/AEM.01541-09 PMC2786419

[42] Segata, N. et al. Metagenomic biomarker discovery and explanation. Genomebiology.com (London. Print) 12 (6): R60-R60. http://genomebiology.com/2011/11/6/R60 (2011). 10.1186/gb-2011-12-6-r60 PMC3218848 21702898

[43] Yuan, M. M. et al. Climate warming enhances microbial network complexity and stability. *Nat. Clim. Change.* **11**(4), 343–348. 10.1038/s41558-021-00989-9 (2021).

[44] Xu, W. F. et al. Structure and ecological function of the soil microbiome associated with ‘Sanghuang’ mushrooms suffering from fungal diseases. *BMC Microbiol.* **23**(1), 218. 10.1186/s12866-023-02965-z (2023). 37573330 10.1186/s12866-023-02965-z PMC10422728

[45] Richard, M. C., David, W. H. & Joseph, W. K. Rhizobacterial colonization of bermudagrass by *Bacillus* spp. in a Marvyn loamy sand soil. *Appl. Soil Ecol.* **141**, 10–17. 10.1016/j.apsoil.2019.04.018 (2019).

[46] Zhang, Q. X. et al. Wheat rhizosphere colonization by *Bacillus**amyloliquefaciens* W10 and *Pseudomonas**protegens* FD6 suppress soil and in planta abundance of the sharp eyespot pathogen *Rhizoctoni*a *cerealis*. *J. Appl. Microbiol.* **134**, 1–11. 10.1093/jambio/lxad101 (2023). 10.1093/jambio/lxad101 37188640

[47] Chen, W. M. et al. Biochar combined with *Bacillus**subtilis* SL-44 as an eco-friendly strategy to improve soil fertility, reduce *Fusarium**wilt*, and promote radish growth. *Ecotoxicol. Environ. Saf.* **251**, 114509. 10.1016/j.ecoenv.2023.114509 (2023). 36621032 10.1016/j.ecoenv.2023.114509

[48] Han, S. C., Kim, K., Maung, C. E. H. & Kim, K. Y. Growth enhancement of tomato by a plant growth promoting bacterium, *bacillus**subtilis* PE7. *Korean J. Soil Sci. Fertil.* **56**(4), 398–406. 10.7745/kjssf.2023.56.4.398 (2023).

[49] Torres, M. et al. Growth promotion on horticultural crops and antifungal activity of *Bacillus**velezensis* XT1. *Appl. Soil Ecol.* **150**, 103453. 10.1016/j.apsoil.2019.103453 (2020).

[50] Shen, Z. Z. et al. Effect of biofertilizer for suppressing Fusarium wilt disease of banana as well as enhancing microbial and chemical properties of soil under greenhouse trial. *Appl. Soil Ecol.* **93**, 111–119. 10.1016/j.apsoil.2015.04.013 (2015).

[51] Wu, L. X., Wang, Y., Lyu, H. & Chen, X. D. Effects of a compound Trichoderma agent on Coptis chinensis growth, nutrients, enzyme activity, and microbial community of rhizosphere soil. *PeerJ* **11**, e15652. 10.7717/peerj.15652 (2023). 37456883 10.7717/peerj.15652 PMC10349559

[52] Asaf, S., Numan, M., Khan, A. L. & Al-Harrasi, A. Sphingomonas: From diversity and genomics to functional role in environmental remediation and plant growth. *Crit. Rev. Biotechnol.* **40**(2), 138–152. 10.1080/07388551.2019.1709793 (2020). 31906737 10.1080/07388551.2019.1709793

[53] Li, Q. et al. Plant growth-promoting rhizobacterium *Pseudomonas* sp. CM11 specifically induces lateral roots. *New Phytol.* **235**(4), 1575–1588. 10.1111/nph.18199 (2022). 35510807 10.1111/nph.18199 PMC9546010

[54] Wang, Q. et al. The endophytic bacterium *Sphingomonas* SaMR12 alleviates Cd stress in oilseed rape through regulation of the GSH-AsA cycle and antioxidative enzymes. *BMC Plant Biol.* **20**, 63. 10.1186/s12870.-020-2273-1 (2020). 32028891 10.1186/s12870-020-2273-1 PMC7006384

[55] Song, J. et al. Rhizosphere microbiomes of potato cultivated under *bacillus**subtilis* treatment influence the quality of potato tubers. *Int. J. Mol. Sci.* **22**(21), 12065–12065. 10.3390/ijms222112065 (2021). 34769506 10.3390/ijms222112065 PMC8584837

[56] Zhao, J. et al. The rhizosphere microbial community response to a bio-organic fertilizer: Finding the mechanisms behind the suppression of watermelon Fusarium wilt disease. *Acta Physiol. Plant.* **40**(1), 17. 10.1007/s11738-017-2581-8 (2017).

[57] Li, X. Y. et al. Microbiome analysis and biocontrol bacteria isolation from rhizosphere soils associated with different sugarcane root rot severity. *Front. Bioeng. Biotechnol.* **10**, 1062351. 10.3389/fbioe.2022.1062351 (2022). 36588942 10.3389/fbioe.2022.1062351 PMC9802638

